# Case report on severe myelin oligodendrocyte glycoprotein antibody-associated disease relapse after ectopic pregnancy and laparoscopic medical abortion: relevance of peripheral inflammation for demyelinating disease activity

**DOI:** 10.3389/fimmu.2025.1582789

**Published:** 2025-04-25

**Authors:** Lidija Smertinaite, Katja Selin, Roosa Vaitiniemi, Ramojus Balevicius, Evangelia Kollia, Tobias Granberg, Maria Isabel Leite, Jacqueline Palace, Helene Blad, Virginija D. Karrenbauer

**Affiliations:** ^1^ Department of Clinical Neuroscience, Karolinska Institutet, Stockholm, Sweden; ^2^ Department of Medicine, Solna, Karolinska Institutet, Stockholm, Sweden; ^3^ Department of Internal Medicine, Gällivare Hospital, Gällivare, Sweden; ^4^ Erasmus+ Exchange Program at Department of Clinical Neuroscience, Karolinska Institutet, Stockholm, Sweden; ^5^ Department of Neuroradiology, Karolinska University Hospital, Stockholm, Sweden; ^6^ Nuffield Department of Clinical Neurosciences, Oxford University, Oxford, United Kingdom; ^7^ Department of Neurology, Nyköpings General Hospital, Nyköping, Sweden; ^8^ Department of Neurology R52, Karolinska University Hospital, Stockholm, Sweden

**Keywords:** magnetic resonance imaging, cerebrospinal fluid, demyelinating disease, myelin oligodendrocyte glycoprotein antibody-associated disease, ectopic pregnancy, multiple sclerosis

## Abstract

**Background:**

Myelin oligodendrocyte glycoprotein antibody-associated disease (MOGAD) is a rare neurological condition. Tubal ectopic pregnancy is an important cause of maternal morbidity and mortality worldwide. Regular pregnancy has a disease-modifying effect on MOGAD, with an increased relapse rate postpartum. Still, there are neither case reports nor cohort studies on abortions and ectopic pregnancy as a disease-modifying event for MOGAD.

**Materials and methods:**

This is a case report on a severe MOGAD relapse after ectopic pregnancy and laparoscopic abortion.

**Discussion:**

For the first time we described that elevated interleukin-1 (IL-1), which was found in cerebrospinal fluid in the current case may be pathogenetically related to ectopic pregnancy. Rituximab (anti-CD20 treatment), downregulated IL-1 and TNF-alfa inflammatory pathways thus is an appropriate drug of choice to treat relapse. Cytokines secreted during ectopic pregnancy could play a disease-modifying role in multiple sclerosis and Guillian-Barré syndrome.

**Conclusion:**

The first case report of a MOGAD severe relapse after ectopic pregnancy and laparoscopic abortion which resolved with rituximab treatment.

## 1 Introduction

Myelin oligodendrocyte glycoprotein antibody-associated disease (MOGAD) is a rare disease, with a prevalence of 20 per million and an incidence of 1.6–3.4 per million people per year (1). According to a systematic review published in 2023, relapse frequency in women with MOGAD declines during normal pregnancy but there is an increased risk of relapse during the postpartum months (2). Meanwhile, though there are multiple case reports and small cohort studies on postpartum outcomes in MOGAD, currently there are no reports on preterm termination of pregnancy or ectopic pregnancy effects on the relapse rate or severity. A study by Landi et al., 2018, showed that in MS patients, abortion was associated with a clinical and radiological rebound effect of 12 months post-event (3). Due to MOGAD being far less prevalent than MS, there is no research published on abortion or ectopic pregnancy as a disease-modifying event in MOGAD; the search in PubMed on November 12th, 2024, gave no results. In the current short report, we review the case of a 43-year-old woman who developed a drastic neuroinflammatory relapse with the phenotype of MOGAD after an ectopic pregnancy and a laparoscopic abortion.

## 2 Case history

A woman who was born in 1976 in the Middle East and moved to Sweden in 2007 was diagnosed with MS in 2010. The patient signed informed consent to participate in the study DNr 2009/2017-31/2 “STOPMS-II”. The study was approved by the Regional Ethical Committee, Stockholm, Sweden. All procedures were performed under the principles of the Declaration of Helsinki. In her medical history, she had a conservatively treated meningioma, hypertension, and gastroesophageal reflux disease. She had no known heredity for neurological diseases. She first presented with optic neuritis in 2008, during her first pregnancy (**Table 1**). McDonalds’ diagnostic criteria for MS were fulfilled in 2009 (**Supplementary Table S1**), (4). A myelin oligodendrocyte glycoprotein (MOG) antibody test was not available at that time. A brain magnetic resonance imaging (MRI) showed nine T2 lesions and an MRI of the spinal cord showed two myelitis lesions. From 2011 to 2014 the patient was treated with interferon beta-1a; expanded disability status scale (EDSS) was 4.0 (2011). Due to relapses and new MRI lesions, the treatment was changed to dimethyl fumarate (DMF) in 2014. In April 2019, an MRI of the brain and spinal cord showed no new lesions (compared to April 2018). The patient was treated with half a dose of DMF since January 2015. The DMF treatment was discontinued in August 2019, as the MRI had not shown any new lesions between April 2018 and April 2019, and the patient was considered to have a secondary-progressive disease course due to EDSS deterioration to 7.5. In addition to impaired gait, she also had urinary incontinence, dysphagia, and deteriorated vision.

**Table 1:** Detailed timeline of the patient’s illness, diagnosis, and treatment.

2008-12-12	The patient presented with suspected optic neuritis and to the neurology clinic. First visit to neurology clinic May 2009.
2009-11-27	McDonalds’ criteria were fulfilled. Brain MRI shows nine T2 lesions. MRI of the spinal cord shows two myelitis lesions. MS diagnosis.
2010-11-25	Treatment with interferon beta-1a started.
2014-10-20	Treatment changed to dimethyl fumarate (DMF) due to side effects.
2015-08-01	DMF treatment changed to half dose due to side effects.
2019-07-12	The patient reported worsening walking ability and urine incontinence.
2019-07-22	The patient came to the emergency department (ED) due to gradual symptom worsening: inability to walk and urine incontinence. ER physician was planning admission to the hospital, but the patient deviated from the ED without explaining the reasons.
2019-08-01	Visit to neurology clinic, out-patient department. DMF treatment was discontinued due to the progression of neurological symptoms; EDSS=8.0.
2019-08-02	The patient sought emergency care and was diagnosed with tubal pregnancy.
2019-08-03	A salpingectomy was performed with no gynecological complications, sHCG=7600 IE/L before surgery.
2019-10-29	MRI of the brain, cervical and thoracic spinal cord was performed without Gadolinium, showing 55 new T2 lesions.
2019-11-22	MRI of the brain and spinal cord showed 8 new gadolinium-enhancing (Gd+) lesions and polyradiculitis in cauda equina.
2019-11-25	CSF analysis: Neurofilament Light=13700 ng/L (ref<890), CXCL13 = 33 ng/L (ref< 7.9), interleukin-1B=17.6 (ref <5.0), presence of oligoclonal bands in CSF.
2019-11-29	The patient received the first rituximab treatment – 1000 mg.
2019-12-09	The CSF sample shows positivity for MOG antibodies. The International MOGAD Panel’s proposed criteria (1) A+B+C were fulfilled.
2020-02-06	Visit to neurology clinic: patient condition improved: patient could take few steps without aid EDSS=7.0.
2020-06-24	MRI of the brain and spinal cord showed no new lesions.
2021-03-23	MRI of the brain and spinal cord showed no new lesions.
2023-02-08	MRI of the brain and spinal cord showed no new lesions.
2024-08-20	MRI of the brain and spinal cord showed no new lesions.

CSF, Cerebrospinal fluid; DMF, Dimethyl fumarate; ED, Emergency department; EDSS, Expanded disability status scale; ER, Emergency room; Gd, Gadolinium; MRI, Magnetic resonance imaging; MS, Multiple sclerosis; sHCG, Serum human chorionic gonadotropin.

In August 2019, the patient sought emergency care for lower abdominal pain and vaginal bleeding. She was diagnosed with tubal pregnancy, with increased levels of serum chorionic gonadotropin 7600 IE/L indicating pregnancy at week 6. A salpingectomy was performed with no gynecological complications.

A month before the abortion, the patient had experienced a worsening of her gait: she could no longer take any steps or stand up without falling. The EDSS was 8.0.

**Figure 1:**
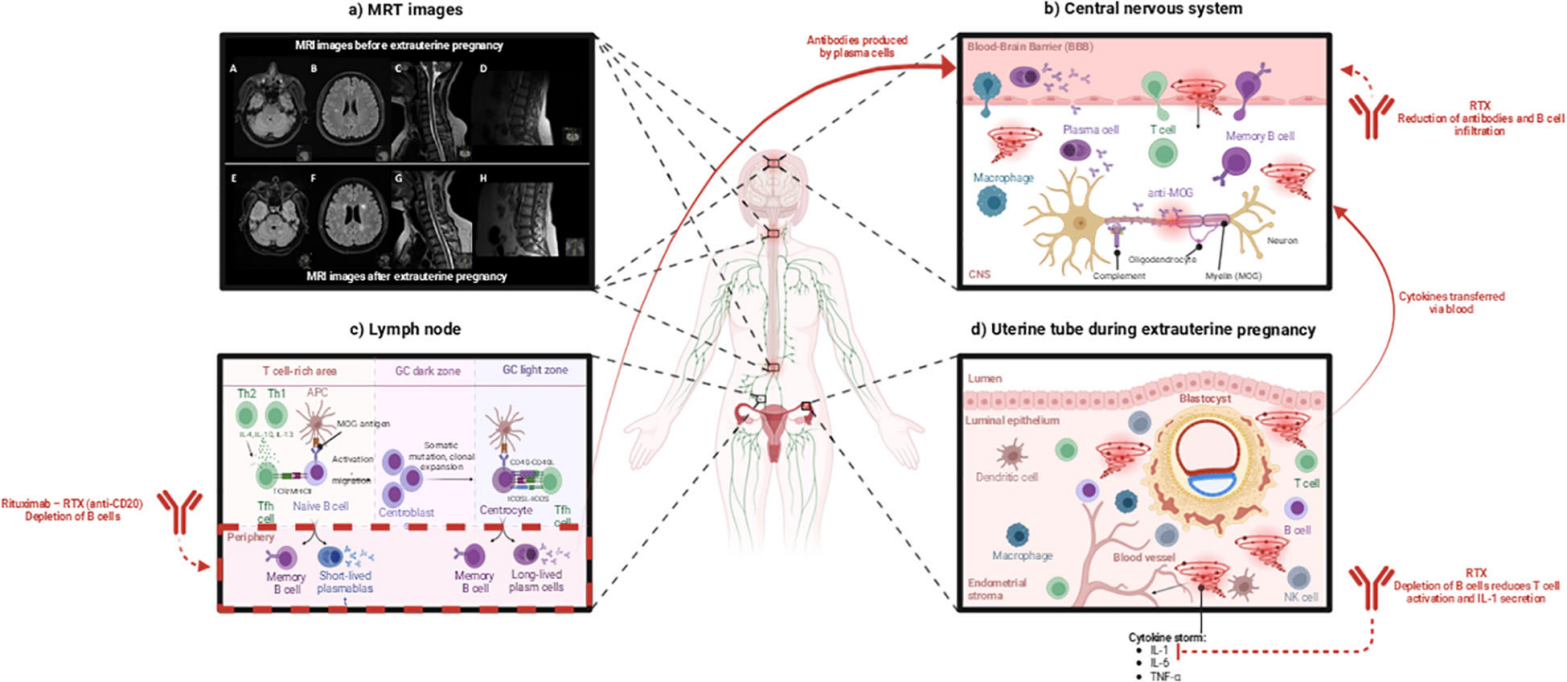
Mechanism of ectopic pregnancy-induced cytokine storm contributing to MOGAD relapse and the therapeutic role of Rituximab (RTX). This image was created with BioRender (https://biorender.com/). **(a)** MRI images before and after ectopic pregnancy. MRI images of the brainstem and cerebellum (A, E), centrum semiovale (B, F), cervical spinal cord at the C1–C2 and C3–C4 levels (C, G), and conus medullaris (D, H) before and after ectopic pregnancy. High-resolution images are provided in the **Supplementary Materials**, **Supplementary Figure S1**. **(b)** Ectopic pregnancy causes a cytokine storm that increases blood-brain barrier (BBB) permeability. Attracted peripheral immune cells and anti-MOG antibodies cross the BBB, infiltrate the central nervous system (CNS), and cause potential damage to neurons [according to Lerch et al., (5)]. RTX plays a crucial role in this process by depleting B cells responsible for producing antibodies and causing CNS damage. **(c)** In the lymph nodes, naive B cells capture the antigen presented by antigen-presenting cells (APCs). The antigen activates the B cell receptor (BCR), which is internalized and presented to T follicular helper (Tfh) cells. This interaction leads to the production of short-lived plasmablasts and memory B cells. The activated B cells enter the germinal center (GC), where they undergo somatic hypermutation and clonal expansion. B cells cycle between the dark and light zones of the germinal center. Antigen-presenting cells (APCs) and Tfh cells help high-affinity B cells survive and differentiate into memory B cells and long-lived plasma cells [according to Stathopoulos & Dalakas, (6)]. The treatment with RTX depletes B cells. **(d)** During an ectopic pregnancy, the blastocyst implants in the uterine tube, triggering an immune cell response and a cytokine storm [according to Mor et al., (7)]. RTX depletes B cells, thus reducing T-cell activation, suppressing IL-1 secretion, and decreasing the cytokine storm-caused immune-mediated tissue damage. APC, Antigen-presenting cell; BBB, Blood-brain barrier; CNS, Central nervous system; MOG, Myelin oligodendrocyte glycoprotein; NK, Natural killer cell; RTX, Rituximab; Tfh, T follicular helper cell; Th1, T helper cell type 1; Th2, T helper cell type 2.

An MRI of the brain in October 2019 showed over 50 new T2 lesions (no gadolinium was administered). In November 2019, a brain MRI showed 8 new gadolinium-enhancing (Gd+) lesions, and MRI of the spinal cord showed Gd+ lesions at levels C2-C3, C5-C7, Th4-Th6, Th8/9, Th11-12 (**Figure 1a**), section C and high-resolution image **Supplementary Figure S1**, section C).

In November 2019, CSF analysis showed a normal count of leukocytes at 5x10^6/L, very high levels of neurofilament light chain (NFL) at 13700 ng/L, the presence of MOG antibodies in CSF and increased levels of interleukin-1 (17.5 ng/L, ref <5 ng/L). Oligoclonal bands (OCB) in CSF had turned positive in 2019. Molecular diagnostic of CSF was negative for Enteroviruses, Epstein Barr virus (0.00 copies/mL), Cytomegalovirus (DNA quantification, 0.00 IU/mL), Herpes simplex virus (HSV)1, HSV2 and Varicella-zoster virus. A complete list of CSF analyses is presented in **Supplementary Materials** (**Supplementary Table S2**) and blood cell count results are presented in **Supplementary Table S3**. The test for serum MOG antibodies (fixed assay) was negative in 2020 and 2023.

Retrospectively, MOGAD diagnostic criteria (1) were fulfilled since December 2019 (**Table 1**).

The patient was treated with RTX, 1000 mg i/v at the end of November 2019, i.e. 4 months after medical abortion. During follow-up clinical visit 3 months after RTX infusion, EDSS improved from 8.0 to 7.0, i.e., the patient could stand and take a few steps. No new lesions have been identified during MRI follow-ups 2021, 2023 and 2024. Serum NFL was normal in February 2023.

## 3 Patient perspective

The patient has been endorsing this publication to spread knowledge on ectopic pregnancy and risks for clinical and radiological worsening in female MOGAD patients.

## 4 Discussion

Both pregnancy and post-partum are marked by significant changes in hormonal levels and consequent immune system adaptations which can in turn affect the course of antibody-mediated disorders (8, 9). Studies have marked reduction in relapse rates for both MOGAD and MS during regular pregnancies as it induces a marked change in both pro- and anti-inflammatory markers of the immune system (8, 10, 11). However, relapses later become more common as the immune system tries to re-establish previous equilibrium in the post-partum period. Here studies conflict whether the rates remain reduced, as some studies have marked a decrease in relapse risk compared to regular rates, while other reports suggest that the postpartum state had higher risk or even facilitated relapses (9, 11).

The severe neuroinflammatory relapse could have been triggered by the many neuroinflammatory factors that are found within ectopic pregnancy and the subsequent abortion; during the transition from late pregnancy to the postpartum period, the immune system goes through major changes, often causing relapse in Th1 and Th17-type autoimmune disorders such as MOGAD (**Figure 1**), (7, 12).

During an ectopic pregnancy (**Figure 1d**), several cytokines are released as part of the body’s response – interleukin-1β (IL-1B), interleukin-6 (IL-6), Tumor Necrosis Factor-alpha (TNF-α), interleukin-8 (IL-8), and Vascular Endothelial Growth Factor (VEGF) (13, 14).

Interleukin-1β has been associated with ectopic pregnancies as well as implicated in pre-term labor or pregnancy complications (13). In the current case, IL-1 was found to be elevated in the patient’s CSF at lumbar puncture performed 17 weeks post-ectopic pregnancy and abortion (**Supplementary Materials**, **Supplementary Table S2**). IL-1B was also found to be increased in the acute demyelinating stage of MOGAD, where it also affects the permeability of the blood-brain barrier (BBB) (**Figure 1b**), (5, 15).

Interleukin-6 was found to be significantly increased in women with tubal ectopic pregnancy and serves with moderate accuracy as a predictor of tubal ectopic pregnancy (16). Increased levels of IL-6 have also been observed in serum and CSF during MOGAD relapses, which contributes to the permeability of the BBB (17, 18).

Interleukin-8 plays a role in attracting neutrophils to sites of inflammation. An elevated level of IL-8 and the subsequent accumulation of neutrophils can lead to tissue damage and inflammation (18). Increased neutrophil/lymphocyte ratio is typical for MOGAD and is a potential biomarker to differentiate MOGAD from MS (19).

TNF-α is increased in the CSF of MOGAD patients during relapses (12). During an ectopic pregnancy, TNF-α can contribute to pain and tissue damage as a powerful pro-inflammatory cytokine (7). However, TNF-α can also affect BBB permeability as it plays a role in leukocyte adhesion, which could facilitate a relapse since acute MOGAD attacks involve T cells and MOG antibodies cross the BBB (**Figure 1c**), (12).

Lastly, VEGF, which is primarily known for its role in angiogenesis, and the formation of new blood vessels, plays an important role in embryogenesis. During ectopic pregnancies, levels of VEGF can be elevated in the body’s effort to support an abnormal implantation site. This can contribute to the inflammatory environment by increasing vascular permeability and promoting the migration of immune cells to sites of inflammation, adding to the previously discussed inflammatory burden (14).

All the inflammatory cytokines, discussed above, are related to ectopic pregnancy and could potentially worsen the inflammatory response in MOGAD, contributing to relapse in different pathogenic ways.

RTX treatment reduces IL-1β and TNF-α expression in peripheral blood mononuclear cells (PBMCs) (6, 20). This reduction occurs through the depletion of CD20+ B cells, which function as antigen-presenting cells (APCs) and play a role in T cell activation. With fewer activated T cells, proinflammatory cytokine signaling decreases downstream, resulting in lower IL-1β and TNF-α production by monocytes and macrophages. The reduction in proinflammatory cytokines helps eliminate neuroinflammation in the cerebrospinal fluid (CSF).

Cytokines secreted during ectopic pregnancy could play a disease-modifying role in multiple sclerosis (MS) and Guillian-Barré syndrome (GBS). According to a study by Nyati et al. (21), in GBS the expression of IL-1, TNF-α, IL-6, and IL-10 was upregulated during the active progressive GBS phase in a case-control study, that included 65 GBS patients (21). In MS serum IL-1 levels correlate with the disease progression in relapse onset MS (22) and serum IL-6 concentration was found to be positively correlated with the MS relapse number in female patients (22). Levels of TNF-α correlated with the degree of disability in patients with progressive MS (23). Abortion in MS patients is associated with inflammatory MS reactivation (22) and the risk of GBS relapse increases after delivery (24) due to surgery and anesthesia may trigger pro-inflammatory cytokines elevation in serum in the postpartum period.

This case report presents a detailed investigation of CSF biomarkers associated with relapse in a patient with MOGAD triggered by ectopic pregnancy. Notably, this is the first report to document elevated IL-1 levels in the CSF in such a context. Additionally, we provide an in-depth discussion of the therapeutic targets of RTX, specifically IL-1 and TNF-α, in the management of MOGAD relapse associated with ectopic pregnancy. Interestingly, similar relapses of MOGAD have also been observed in other contexts, such as following SARS-CoV-2 infection, highlighting potential shared mechanisms that may inform treatment strategies (25).

## 5 Conclusion

This case report illustrates that ectopic pregnancy and medical abortion could be potential disease-modifying events for the MOGAD. IL-1 in CSF was identified as a possible abortion-event-related cytokine, that was implicated in MOGAD relapse. RTX was an effective treatment to resolve abortion-related MOGAD inflammatory relapse by targeting IL-1 and TNF-α inflammatory pathways.

## Data Availability

The original contributions presented in the study are included in the article/**Supplementary Material**. Further inquiries can be directed to the corresponding author.

## References

[1] Banwell B Bennett JL Marignier R Kim HJ Brilot F Flanagan EP. Diagnosis of myelin oligodendrocyte glycoprotein antibody-associated disease: International MOGAD Panel proposed criteria. Lancet Neurol. (2023) 22:268–82. doi: 10.1016/S1474-4422(22)00431-8 36706773

[2] Leite MI Panahloo Z Harrison N Palace J. A systematic literature review to examine the considerations around pregnancy in women of child-bearing age with myelin oligodendrocyte glycoprotein antibody-associated disease (MOGAD) or aquaporin 4 neuromyelitis optica spectrum disorder (AQP4+ NMOSD). Mult Scler Relat Disord. (2023) 75:104760. doi: 10.1016/j.msard.2023.104760 37224631

[3] Landi D Ragonese P Prosperini L Nociti V Haggiag S Cortese A. Abortion induces reactivation of inflammation in relapsing-remitting multiple sclerosis. J Neurol Neurosurg Psychiatry. (2018) 89:1272–8. doi: 10.1136/jnnp-2018-318468 29970387

[4] Polman CH Reingold SC Edan G Filippi M Hartung HP Kappos L. Diagnostic criteria for multiple sclerosis: 2005 revisions to the “McDonald Criteria. Ann Neurol. (2005) 58:840–6. doi: 10.1002/ana.20703 16283615

[5] Lerch M Bauer A Reindl M. The potential pathogenicity of myelin oligodendrocyte glycoprotein antibodies in the optic pathway. J Neuroophthalmol. (2023) 43:5–16. doi: 10.1097/WNO.0000000000001772 36729854 PMC9924971

[6] Stathopoulos P Dalakas MC. Evolution of anti-B cell therapeutics in autoimmune neurological diseases. Neurotherapeutics. (2022) 19:691–710. doi: 10.1007/s13311-022-01196-w 35182380 PMC9294112

[7] Mor G Aldo P Alvero AB. The unique immunological and microbial aspects of pregnancy. Nat Rev Immunol. (2017) 17:469–82. doi: 10.1038/nri.2017.64 28627518

[8] Bränn E Edvinsson Å Rostedt Punga A Sundström-Poromaa I Skalkidou A. Inflammatory and anti-inflammatory markers in plasma: from late pregnancy to early postpartum. Sci Rep. (2019) 9:1863. doi: 10.1038/s41598-018-38304-w 30755659 PMC6372606

[9] Cortese R Mariotto S Mancinelli CR Tortorella C. Pregnancy and antibody-mediated CNS disorders: What do we know and what should we know? Front Neurol. (2022) 13:1048502. doi: 10.3389/fneur.2022.1048502 36601293 PMC9806181

[10] Dobson R Jokubaitis VG Giovannoni G. Change in pregnancy-associated multiple sclerosis relapse rates over time: a meta-analysis. Mult Scler Relat Disord. (2020) 44:102241. doi: 10.1016/j.msard.2020.102241 32521483

[11] Carra-Dallière C Rollot F Deschamps R Ciron J Vukusic S Audoin B. Pregnancy and post-partum in patients with myelin-oligodendrocyte glycoprotein antibody-associated disease. Mult Scler Houndmills Basingstoke Engl. (2023) 29:270–6. doi: 10.1177/13524585221134214 36453174

[12] Corbali O Chitnis T. Pathophysiology of myelin oligodendrocyte glycoprotein antibody disease. Front Neurol. (2023) 14:1137998. doi: 10.3389/fneur.2023.1137998 36925938 PMC10011114

[13] Lekovich J Witkin SS Doulaveris G Orfanelli T Shulman B Pereira N. Elevated serum interleukin-1β levels and interleukin-1β-to-interleukin-1 receptor antagonist ratio 1 week after embryo transfer are associated with ectopic pregnancy. Fertil Steril. (2015) 104:1190–4. doi: 10.1016/j.fertnstert.2015.07.1145 26279136

[14] Cabar FR Pereira PP Oliveira MA Francisco RPV. Serum vascular endothelial growth factor as a marker for tubal pregnancy. Rev Assoc Med Bras (1992). (2022) 68:860–5. doi: 10.1590/1806-9282.20220224 PMC9575910 35766702

[15] O’Carroll SJ Kho DT Wiltshire R Nelson V Rotimi O Johnson R. Pro-inflammatory TNFα and IL-1β differentially regulate the inflammatory phenotype of brain microvascular endothelial cells. J Neuroinflammation. (2015) 12:131. doi: 10.1186/s12974-015-0346-0 26152369 PMC4506411

[16] Bickel M. The role of interleukin-8 in inflammation and mechanisms of regulation. J Periodontol. (1993) 64:456–60. 8315568

[17] Rajendiran S Senthil Kumar GP Nimesh A Dhiman P Shivaraman K Soundararaghavan S. Diagnostic significance of IL-6 and IL-8 in tubal ectopic pregnancy. J Obstet Gynaecol. (2016) 36:909–11. doi: 10.1080/01443615.2016.1174821 27612507

[18] Shimizu F Nakamori M. Blood-brain barrier disruption in neuroimmunological disease. Int J Mol Sci. (2024) 25. doi: 10.3390/ijms251910625 PMC11476930 39408955

[19] Lin L Ji M Wu Y Hang H Lu J. Neutrophil to lymphocyte ratio may be a useful marker in distinguishing MOGAD and MS and platelet to lymphocyte ratio associated with MOGAD activity. Mult Scler Relat Disord. (2023) 71:104570. doi: 10.1016/j.msard.2023.104570 36827875

[20] Tavakolpour S Alesaeidi S Darvishi M GhasemiAdl M Darabi-Monadi S Akhlaghdoust M. A comprehensive review of rituximab therapy in rheumatoid arthritis patients. Clin Rheumatol. (2019) 38:2977–94. doi: 10.1007/s10067-019-04699-8 31367943

[21] Nyati KK Prasad KN Rizwan A Verma A Paliwal VK. TH1 and TH2 response to Campylobacter jejuni antigen in Guillain-Barre syndrome. Arch Neurol. (2011) 68:445–52. doi: 10.1001/archneurol.2011.51 21482924

[22] de Jong BA Huizinga TW Bollen EL Uitdehaag BM Bosma GP van Buchem MA. Production of IL-1beta and IL-1Ra as risk factors for susceptibility and progression of relapse-onset multiple sclerosis. J Neuroimmunol. (2002) 126:172–9. doi: 10.1016/S0165-5728(02)00056-5 12020968

[23] Chen YC Yang X Miao L Liu ZG Li W Zhao ZX. Serum level of interleukin-6 in Chinese patients with multiple sclerosis. J Neuroimmunol. (2012) 249:109–11. doi: 10.1016/j.jneuroim.2012.04.015 22633195

[24] Cheng Q Jiang GX Fredrikson S Link H de Pedro-Cuesta J. Increased incidence of Guillain-Barré syndrome postpartum. Epidemiology. (1998) 9:601–4. doi: 10.1097/00001648-199811000-00006 9799167

[25] Woodhall M Mitchell JW Gibbons E Healy S Waters P Huda S. Case report: myelin oligodendrocyte glycoprotein antibody-associated relapse with COVID-19. Front Neurol. (2020) 11:598531/BIBTEX. doi: 10.3389/FNEUR.2020.598531/BIBTEX 33324337 PMC7724101

