# Supplementary material for: Case report on severe myelin oligodendrocyte glycoprotein antibody-associated disease relapse after ectopic pregnancy and laparoscopic medical abortion: relevance of peripheral inflammation for demyelinating disease activity
Source: Front Immunol. 2025 Apr 25;16:1582789. doi: 10.3389/fimmu.2025.1582789 (PMC12061928; doi:10.3389/fimmu.2025.1582789)
Supplement: Supplementary file 1 [file Table1.docx]

Supplementary Material

# **Supplementary Tables**

**Table S1.** Timeline of the fulfillment of myelin oligodendrocyte glycoprotein antibody-associated disease (MOGAD) diagnostic criteria, Banwell, et al., 2018.

| MOGAD diagnostic criteria | | Criteria Year | 2008 December | 2009 may | 2009 November | | 2015  January | 2018  April | 2019  October | 2019  December | 2021 | 2024 |
| --- | --- | --- | --- | --- | --- | --- | --- | --- | --- | --- | --- | --- |
|  |  | (A) Core clinical demyelinating event (optic neuritis, myelitis, ADEM) | Retrobulbar ON left | Bilateral optic nerve atrophy | New cerebellar lesion  New pons lesion | | Yes | Yes | Myelitis | Myelitis | Yes | Yes |
|  |  | (B) Positive MOG-IgG test (clear positive, or low positive or negative but CSF-positive with supporting clinical or MRI features | N/A | N/A | N/A | | N/A | N/A | N/A | Yes* | Yes | Yes |
|  |  | Supportive Clinical or MRI features (optic neuritis, myelitis, brain, brainstem or cerebral syndrome) | N/A | N/A | Fluffy lesions | | BrainstemFluffy lesions,  H-sign | Fluffy lesions | Cortical lesions, fluffy lesions, LETM | LETM,  Conus medullaris lesions |  |  |
|  |  | (C) Exclusion of better diagnoses including multiple sclerosis | No | No | No | | No | No | No | Yes | Yes | Yes |
|  |  | A, B, and C criteria fulfilled | A | A | A+B | | A+B | A+B | A+B | A+B+C | A+B+C | A+B+C |
|  | | MOG-AD diagnosis | No | No | No | | No | No | No | Yes | Yes | Yes |
| McDonalds Criteria | | DIS | No | No  (1 PV lesion) | Yes (3 T2 lesions: PV, Cer, pons) | | Yes | Yes | Yes | Yes | Yes | Yes |
|  |  | DIT | No | No | Yes (I Gd+) | | Yes | Yes | Yes | Yes | Yes | Yes |
| MS Diagnosis | | | No | No | Yes | | Yes | Yes | Yes | No  (Conus medullaris lesions and MOG AB in CSF excludes MS diagnosis) | No | No |
| CSF  findings | | OCB | N/A | 2010 may IgG index normal  No OCBs | | | | | 2019 November  OCB positive | | | |
|  |  | KFLC | N/A | N/A | | | | | Positive | | | |
|  |  | MOG AB | N/A |  |  |  |  |  | Positive | | | |
| AQP MOG AB in serum | | | N/A | N/A | | | | | Negative | | | |
| Radiological progress | | |  |  | Yes | | Yes  1 Gd+ |  | >50 T2 lesions | 8 New Gd+ lesions | No | No |
| Treatment | MS | N/A | N/A | N/A | | Interferon beta-1a 2011-2015 | | DMF January 2015 – August 2019 | | Rituximab 1000 mg i/v | Treatment free | |
|  | MOG-AD | N/A | N/A | N/A | N/A | | N/A | N/A | N/A |  |  |  |

Abbreviations: ADEM = Acute Disseminated Encephalomyelitis; AQP = Aquaporin; Cer = Cerebral; CSF = Cerebrospinal fluid; Gd+ = Gadolinium; IgG = Immunoglobulin G; KFLC = Kappa free light chains; LETM = Longitudinally Extensive Transverse Myelitis; MOG AB = myelin oligodendrocyte glycoprotein antibody; MOGAD = myelin oligodendrocyte glycoprotein antibody-associated disease; MRI = Magnetic resonance imaging; MS = Multiple sclerosis; N/A = Not Available; OCB = Oligoclonal bands; ON = Optic neuritis; PV = Periventricular. * = MOG-IgG test negative in serum but CSF-positive with supporting MRI features.

**
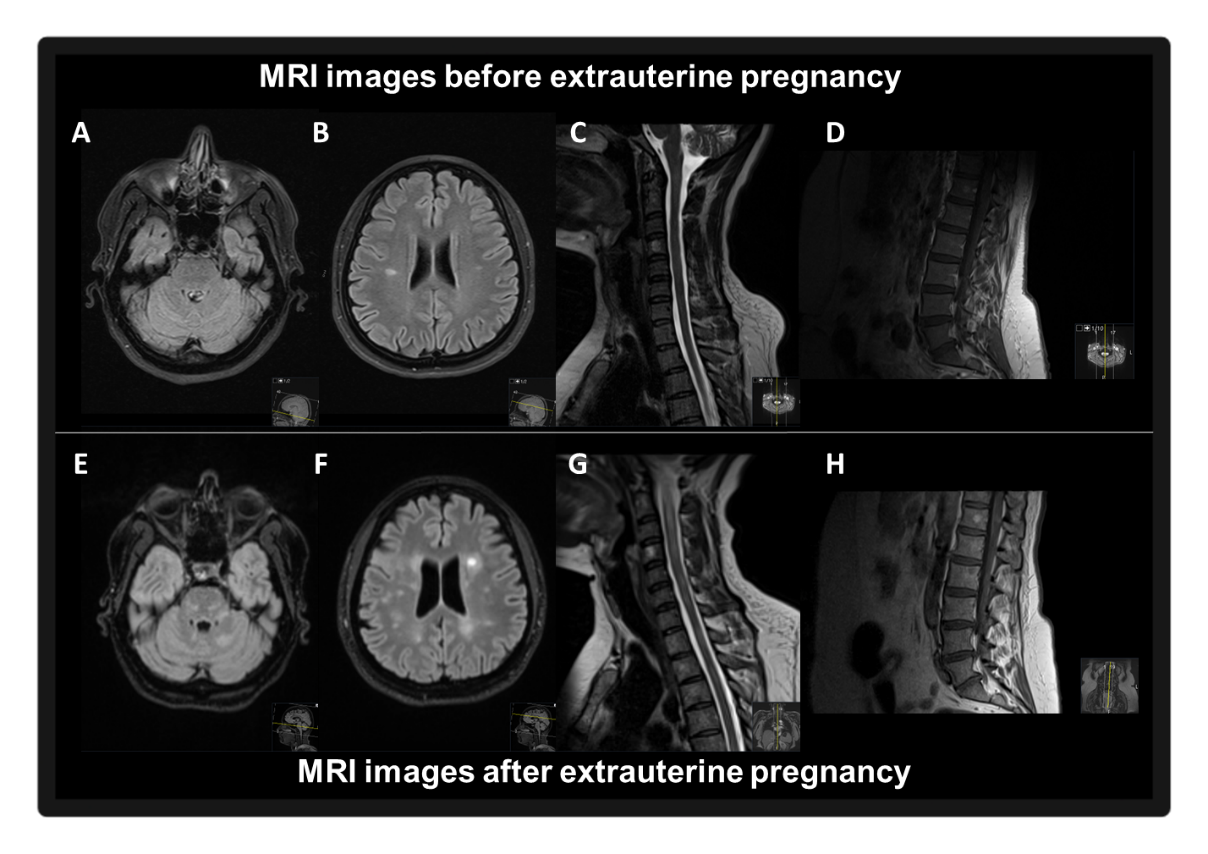
**

**Figure S1.** MRI images before and after ectopic pregnancy. MRI images of the brainstem and cerebellum (A, E), centrum semiovale (B, F), cervical spinal cord at the C1–C2 and C3–C4 levels (C, G), and conus medullaris (D, H) before and after ectopic pregnancy.

**Table S2.** Cerebrospinal fluid analyses in 2010 and 2019.

| **Analysis (unit)** | **2010**  **May** | **2019 November** | **Reference interval** |
| --- | --- | --- | --- |
| CSF Albumin (mg/L) | 296 | 351 | <280*, <320** |
| CSF/S-Albumin ratio (${\times10}^{-3}$) | 7.0 | 10.0 | <7.0 |
| CSF IgG (mg/L) | 56 | 56 | <45 |
| CSF IgG index | 0.58 | 0.48 | <0.70 |
| CSF leukocytes ($\times{10}^{6}$/L) | N/A | 5.0 | 0.0-5.0 |
| CSF erythrocytes ($\times{10}^{6}$/L) | N/A | 1.0 | <1.0 |
| CSF FLC-K (mg/L) | N/A | 1.24 | <0.34 |
| CSF KFLC-IF | N/A | 0.35 | <0.00 |
| CSF AQP-4 antibodies | N/A | Negative | Negative |
| CSF NFL (ng/L) | N/A | 13 700 | <890 |
| CSF MOG antibodies | N/A | Positive**^#^** | Negative |
| CSF OCB | Absent | Present | Absent |
| CSF CXCL13 (ng/L) | N/A | 33 | <7.8 |
| CSF Interleukin-1 (ng/L) | N/A | 17.6 | <5.0 |

Abbreviations: AQP = Aquaporin; CSF = Cerebrospinal fluid; CXCL13 = C-X-C motif chemokine 13; IgG = Immunoglobulin G; KFLC = Kappa free light chains; L = liter; mg = milligram; MOG = myelin oligodendrocyte glycoprotein; N/A = Not available; ng = nanogram; OCB = Oligoclonal bands; S = Serum; **^#^** = analysis was performed December 2019; * = Reference values in 2010; ** = Reference values in 2019.

**Table S3.** Peripheral blood test in August and November 2019.

| **Blood cell count (unit)** | **2019**  **August** | **2019**  **November** | **Reference**  **interval** |
| --- | --- | --- | --- |
| Leukocytes (10^9^/L) | 5.9 | 5.7 | 3.5-8.8 |
| Erythrocytes (10^12^/L) | 4.9 | 4.5 | 3.9-5.2 |
| Thrombocytes (10^9^/L) | 297 | 277 | 165-387 |
| Neutrophils (10^9^/L) | N/A | 3.8 | 1.6-5.9 |
| Eosinophils (10^9^/L) | N/A | 0.3 | 0.0-0.5 |
| Basophils (10^9^/L) | N/A | <0.1 | 0.0-0.1 |
| Monocytes (10^9^/L) | N/A | 0.4 | 0.2-0.8 |
| Lymphocytes (10^9^/L) | N/A | 1.1 | 1.1-3.5 |
| T lymphocytes (CD3+) (10^9^/L) | N/A | 0.47 | 0.78-2.07 |
| B lymphocytes (CD19+) (10^9^/L) | N/A | 0.17 | 0.09-0.4 |
| NK cells (CD16-/CD56+/CD3-) (10^9^/L) | N/A | 0.07 | 0.07-0.42 |

Abbreviations: N/A = Not Available.
